# Insight into novel clinical mutants of RpsA-S324F, E325K, and G341R of *Mycobacterium tuberculosis* associated with pyrazinamide resistance

**DOI:** 10.1016/j.csbj.2018.09.004

**Published:** 2018-10-04

**Authors:** Muhammad Tahir Khan, Ashfaq Ur Rehaman, Muhammad Junaid, Shaukat Iqbal Malik, Dong-Qing Wei

**Affiliations:** a Department of Bioinformatics and Biosciences, Capital University of Science and Technology, Pakistan.; b College of Life Sciences and Biotechnology, The State Key Laboratory of Microbial Metabolism, Shanghai Jiao Tong University, China

**Keywords:** Mutations, RpsA, POA, Simulations, Resistance, RMSD

## Abstract

Pyrazinamide (PZA) is an important component of first-line anti-tuberculosis drugs which is converted into active form, pyrazinoic acid (POA), by *Mycobacterium tuberculosis* (MTB) *pncA* gene encoded, pyrazinamidase (PZase). Mutations in *pncA* are detected in >70% of PZA resistant isolates but, noticeably, not in all. In this study, we selected 18 PZA-resistant but wild type *pncA* (pncA^WT^) MTB isolates. Drug susceptibility testing (DST) of all the isolates were repeated at the critical concentration of PZA drug. All these PZA-resistance but pncA^WT^ isolates were subjected to RpsA sequencing. Fifteen different mutations were identified in eleven isolates, where seven were present in a conserved region including, Ser324Phe, Glu325Lys, Gly341Arg. As the molecular mechanism of resistance behind these variants has not been reported earlier, we have performed multiple analysis to unveil the mechanisms of resistance behind mutations S324F, E325K, and G341R. The mutant and wild type RpsA structures were subjected to comprehensive computational molecular dynamic simulations at 50 ns. Root mean square deviation (RMSD), Root mean square fluctuation (RMSF), and Gibbs free energy of mutants were analyzed in comparison with wild type. Docking score of wild type*-*RpsA has been found to be maximum, showing a strong binding affinity in comparison with mutants. Pocket volume, RMSD and RMSF have also been found to be altered, whereas total energy, folding effect (radius of gyration) and shape complimentarily analysis showed that variants S324F, E325K, and G341R have been playing a significant role behind PZA-resistance. The study offers valuable information for better management of drug resistance tuberculosis.

## Graphical abstract

**Figure:**
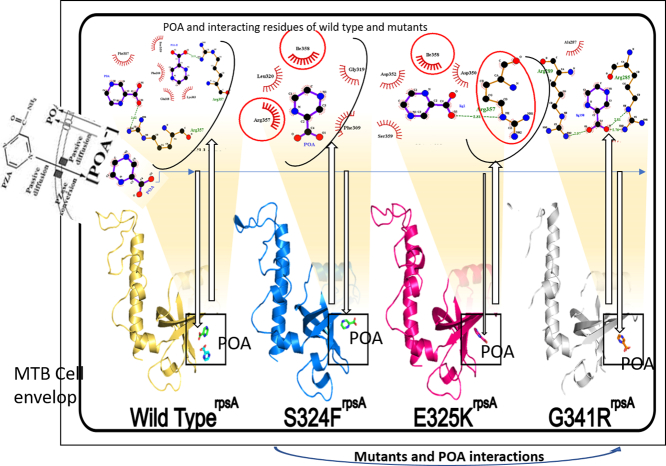

## 1 Introduction

Pyrazinamide (PZA) is a first-line drug used in combination with rifampin and isoniazid that kills persister bacilli, and was found to be effective in shortening the duration of TB therapy [1,2]. The prodrug is converted into an active state, pyrazinoic acid (POA), by *Mycobacterium tuberculosis* (MTB) *pyrazinamidase (PZase*) encoded by *pncA*. The major targets of POA that have so far been identified are ribosomal protein S1 (RpsA), which is involved in trans-translation, and aspartate decarboxylase (*panD*), which is involved in ATP synthesis [3,4]. Shi et al., confirmed POA binds with RpsA, disrupting the formation of the RpsA–tmRNA complex [5], while POA is unable to bind mutant RpsA that results in POA resistance. Deleting Alanine (RpsA_ΔA438_) at the C-terminal showed PZA resistance and lack of binding to RpsA in *Mycobacterium smegmatis*. Shi et al., concluded that the C-terminal region of RpsA is the interaction site of POA as it interferes with the transfer-messenger RNA (tmRNA) complex formation during initiation of translation.

In bacteria, *Escherichia coli,* encoded RpsA there are six S1 domains, whereas MTB has four S1 domains [6]. The first two domains interact and are involved in ribosomal binding and the last two may be involved to bind RNA (Salah et al., 2009). Bycroft et al., identified some conserved amino acids, Phe307, Phe310, His322, Asp352, and Arg357 as RNA binding sites [7]. Yang et al., demonstrated that residues 292–363 forming the fourth S1 domain is highly conserved and fully capable of interaction with POA in mycobacterial species. Mutations at the C-terminus of MTB RpsA (MtRpsA^CTD^) may alter interactions with POA in the fourth S1 domain, leading to conformational changes in the POA binding site resulting drug resistance [2]. Two POA molecules form a complex through Lys303, Phe307, Phe310 and Arg357 making hydrogen and hydrophobic interactions that are essential for tmRNA bindings [2,5].

Here we analyzed the molecular mechanism behind the resistance due to mutations Ser324Phe, Glu325Lys, Gly341Arg present in the conserved region of RpsA. In our previous study [8], these mutations were detected in the rpsA gene of PZA-resistance containing a WT pncA gene. Here, we have addressed the conformational changes that resulted from mutations Ser324Phe, Glu325Lys, and Gly341Arg that may be very helpful in understanding the mechanism behind resistance. We performed the molecular dynamic simulations including interactions of POA with wild type and mutant RpsA to improve understanding and management of drug resistance leading to novel drug design.

## 2 Material and Methods

### 2.1 Study Samples

As described in our previous findings [8], we collected 18 samples from the Provincial Tuberculosis Reference Laboratory (PTRL) that were previously identified as PZA resistance (PZA R) but *pncA*^WT^. PTRL is the only central reference laboratory of Khyber Pakhtunkhwa (KPK) province, receiving samples for drug susceptibility testing.

### 2.2 Drug Susceptibility Testing (DST)

Drug susceptibility testing was performed using the automated BACTEC MGIT 960 system. *Mycobacterium tuberculosis* strain ATCC 25618 / H37Rv and *Mycobacterium bovis* was used as negative and positive controls respectively. Growth at a critical concentration of PZA (100 μg/ml) was considered to indicate PZA-resistance.

### 2.3 DNA Extraction, PCR Amplification and Sequencing

PZA resistant samples were subjected for genomic DNA extraction using the sonication method. [9,10]. The fragments containing RpsA (1544-bp fragment, including the entire RpsA open reading region, 81 bp of the downstream sequence, 17 bp of the upstream sequence) were amplified using the following previously reported primers: RpsA-F (5′CGGAGCAACCCAACAATA-3′), RpsA-R (5′-GTGGACAGCAACGACT TC-3′) [11]. Each 50-μl PCR reaction contained 0.1 μl of each DNTs, 3 μl MgCl_2,_ 5 μl PCR buffer, 0.8 μl Taq (New England Biolabs, UK)), 1 μl each forward and reverse primers, 34.8 μl molecular grade water and 4 μl of genomic DNA. The PCR conditions were set as, 5 min at 94 °C for the denaturation step; 30 cycles of 30 s, 30 s at 56 °C, and 72 °C for 1 min; an extension step at 72 °C for 5 min as previously described. The PCR product was analyzed using 6 Applied Biosystems 3730xl (Macrogen, Korea).

### 2.4 DNA Sequence Analysis

To find mutations behind PZA-resistance, the sequence data were loaded into Mutation Surveyor V5.0.1 and compared with RpsA (Rv1630) from the RefSeq (NC_000962.3) database of NCBI.

### 2.5 Homology Modeling of Mutants of RpsA and Generation of Ligand

A three dimensional structure of MTB RpsA [2] (PDB ID 4NNK) was retrieved from the Brookhaven Raster Display (BRAD) protein data bank (PDB) [12]. Prior to further analysis of RpsA structure, all the water of crystallization was removed. Mutant structure of RpsA was not available in PDB, hence we created mutations at locations, Ser324Phe, Glu325Lys, Gly341Arg using PYMOL [13]. The mutant structures were validated through Ramachandran Plot [14].

The drug POA was retrieved from PubChem (PubChem CID: 1047) [15] and energy minimization was carried in Molecular Operation Environment (MOE) using MMFF94X forcefield [16,17].

### 2.6 Molecular Docking between RpsA Proteins and the Ligand

Protein was prepared using proteins preparation option in MOE. Incorrect hydrogen atoms were corrected while selenomethionines were changed into methionine. Shape complementarity of protein and drug was measured in the form of score using PatchDock server [18]. This process is also known as geometric matching, is a method where receptor and ligand features, like molecular surface is compared to find them dockable. Intermolecular interfaces are a typical phenomenon in biological systems where molecular complexes exhibit high shape complementarity to interact. Ligand and receptor shape complementarity at the interface of their complexes is a logical practice and would work satisfactorily in re-docking. Proteins often undergo conformational changes in order to create a highly complementary interface when associating with a drug [19,20]. The geometric score of the mutant in comparison with the wild type were analyzed. Pocket volumes of wild type and mutant RpsA were compared through CastP server [21]. RpsA-POA interactions were analyzed using Ligplot as described in earlier studies [22,23]**.**

### 2.7 Molecular Dynamics Simulation (MD)

MD simulation was run on all the complexes using AMBER while the PRODRG web server was used to generate the topology of ligand. A cubic box of 1.5 nm was solvated with each complex using a simple point charge (spc) water model [24]. Sodium and chloride ions were added into the cubic box to neutralize the systems. These ions had maximum electrostatic potential replacing the water molecules. Energy minimization was done for 100,000 cycles using the steepest descent algorithm.

Equilibration was carried out with constant temperature, constant volume (NVT) ensemble at 300 K followed by constant temperature, constant pressure (NPT) ensemble for 300 K and 1 bar pressure with each for 100 ps. The Berendsen thermostat method was used for temperature [25] while pressure was maintained constant by Parrinello–Rahman barostat [26]. Bond length was rectified with Linear constraint solver (LINCS) algorithm [27].

### 2.8 Principal Component Analysis

Principal Component Analysis (PCA) of a MD simulation was performed on the mass-weighted cartesian coordinates. Internal motion of the system was obtained by removing the overall rotation and its translation from the trajectory. PCA is carried with long time dynamics by recognizing low modes in proteins [28,29]. PCA reduces and simplifies the complicated movement in long trajectory generated during MD simulation [[30], [31], [32]]. A transformed set of variables z1, z2…, zp called principal components (PCs) were generated during PCA. Energies of sets of macromolecule conformations is described by Free Energy Landscape (FEL) [33,34] . The first two components called PC1 and PC2 give the trajectories on initial two principal components of motion.

### 2.9 The Gibbs Free Energy

The available energy often called Gibbs free energy (G) (Sugita & Kitao, 1998) was plotted against wild type*-*RpsA. The G is minimized at constant pressure and temperature to chemical equilibrium state of system. It is a thermodynamic potential at constant temperature and pressure, where reduction in G is an essential state for the freedom of processes.

## 3 Results

**Fig. 2:**
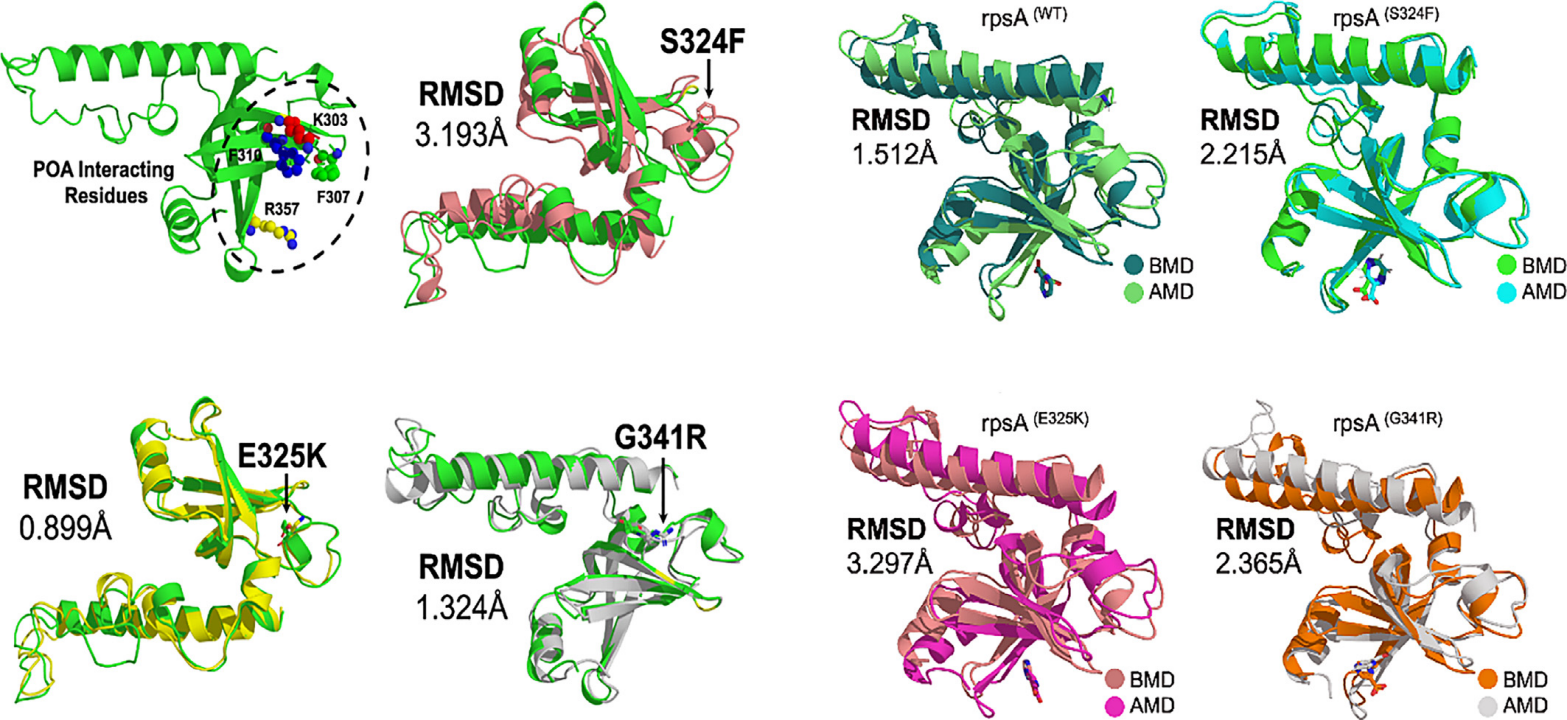
Superimposition of wild type-RpsA (green) and mutant. (a) Structure of MTB RpsA and POA binding residues 303, 307, 310, and 357. (b, c, d) Superimposition of mutant S324F, E325K, and G341R and wild type-RpsA. (A) Superimposition of wild type before and after simulation. (B,C,D) Superimposition of mutants before and after simulation.

Drug susceptibility results show that all the samples were PZA R. Of 18 PZA R isolates, 11 (61%) isolates harbored fifteen different mutations, whereas seven PZA R isolates were RpsA^WT^ (Table 1). Mutations, Ser324Phe, Glu325Lys, Gly341Arg, Asp342Asn, Asp343Asn, Ala344Pro, and Ile351Phe were detected in the conserved region (292–363) of the RpsA gene (Table 1). To explore the molecular mechanism behind resistance, the first three conserved region mutations (S324F, E325K, and G341R) were subjected to MD simulation at 50 ns. We have detected significant changes in structure and activity of RpsA due to mutations S324F, E325K, and G341R. The overall result showed a significant effect of mutant on the RpsA activity. (See Fig. 2.)

**Table 1:** Mutations in RpsA gene in PZA resistant *pncA*^*WT*^ isolates [8].

SNO	Nucleotide Position	Codon No.	Codon Change	Amino Acid Change	Frequency (No. of strains)
1	76delA	26	ATA	Ile26FRAME	1
2	220G > A	74	GTC > ATC	Val74Ile	1
3	278A > G	93	AAG > AGG	Lys93Arg	1
4	618G > A	206	TTG > TTA	Leu206Leu	2
5	636A > C	212	CGA > CGC	Arg212Arg	2
6	830A > G	277	AAG > AGG	Lys277Arg	1
7	971C > T	324	TCC > TTC	Ser324Phe	1
8	973G > A	325	GAG>AAG	Glu325Lys	3
9	1021G > C	341	GGC > CGC	Gly341Arg	1
10	1024G > A	342	GAC > AAC	Asp342Asn	4
11	1027G > A	343	GAC > AAC	Asp343Asn	6
12	1030G > C	344	GCG > CCG	Ala344Pro	6
13	1051A > T	351	ATC > TTC	Ile351Phe	3
14	1108A > C	370	ACC > CCC	Thr370Pro	1
15	1207 T > G	403	TGG > GGG	Trp403Gly	1

### 3.1 Binding Pocket Volume and Shape Complementarity

Shape complementarity score of wild type was found maximum (2352) in comparison with mutants (Table 2). Pocket volume of wild type and mutants were compared through CastP server. The mutants, S324F, E325K, and G341R have a binding pocket volume of 110.424 Å, 501.522 Å, and 563.383 Å seems deviated from wild type, 499.310 Å. This increase or decrease in binding pocket volume may affect the firm interaction of protein with drug. These findings clarify the effect of mutations on RpsA activity.

**Table 2:** Docking score and pocket volume of wild and mutant RpsA.

Protein	PatchDock score	Volume (Å)
Wild type	2352	499.310
S324F	2068	110.424
E325K	1864	501.522
G341R	1952	563.383

### 3.2 Model Validation and Proteins-Ligand Interactions

**Fig. 1:**
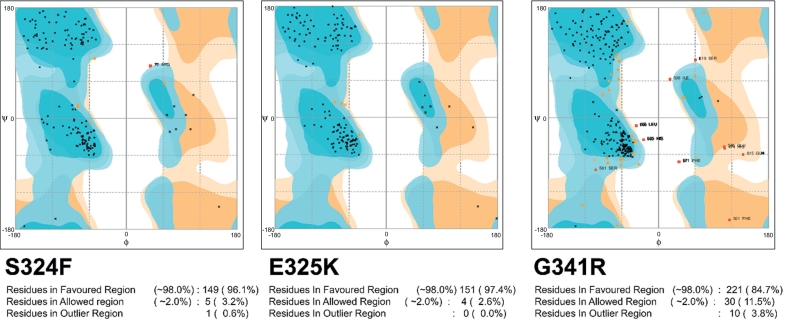
Mutant model validation (ramachandran plot).

The majority of the residues of mutant structure were found in a favorable region (Fig. 1). Number of hydrogen and hydrophobic interactions are important in protein activity. Overall the residues Arg357, Phe309, Gly319, leu320, Ala287, Ileu358, Asp352, Asp350, Ser359, Leu353, Phe307, Phe310, Glu318, and Lys303 were found to be involved in hydrogen and hydrophobic interactions. Wild type protein formed two H-bonds and four hydrophobic with POA. Mutants S324F and E325K had fewer hydrogen and hydrophobic interactions, as shown in figure (Fig. 6).

### 3.3 Protein and Drug Trajectory

A molecular dynamics simulation was run for 50 ns on high performance compunting of the RpsA complex with drug. Wild type structure attained a root mean square deviation (RMSD) of 2.0 Å, 4.8 Å and 1.8 Å at 0 ns, 23 ns, and 50 ns respectively to attain the stability. The mutatnt S324F showed a deviation and attained the RMSD value of 2.1 Å, 1.2 Å, and 4.2 Å at 0 ns, 28 ns and 40 ns respectively, but at 50 ns it attained some stability (3.8 Å). Mutant E325K attained the RMSD between 1.1 Å and 4.8 Å at 9 ns and 45 ns repectively. However, at 50 ns the final RMSD was found to be 3.4 Å, slightly higher than wild type. The RMSD values of G341R were found to be between 1.2 Å and 4.0 at 10 ns and 25 ns repectively. The RMSD seemed to be stable after 35 ns with a final value of 3.2 Å at 50 ns. The deviation in the mutant seemed to be low, but the final value of wild type at 50 ns was found to be lower initially but higher than mutants after some time (Fig. 4).

Fluctuations in mutant S324F, E325K, and G341R residues were a little high in comparison with wild type*-* RpsA. Wild type exhibit RMSF of 0.3–3.8 Å. These fluctuations appeared to be present in between residues 358–400, whereas mutant S324F exhibited 0.4-9 Å where fluctuation was observed between residues 358–434. Mutant E325K has a RMSF value between 0.7 Å and 4.7 Å and exhibited fluctuations in residues 358–434. Mutation at position G341R exhibited fluctuation in between 0.8 and 5.2 Å in which fluctuation was observed in residues 355–434. The RMSF value for S324F, E325K, and G341R demonstrated a higher flexibility, resulting in low affinity for POA. Residues of the conserved area forming the fourth S1 domain may be altered in RpsA (Fig. 5).

### 3.4 Radius of Gyration (Rg)

**Fig. 3:**
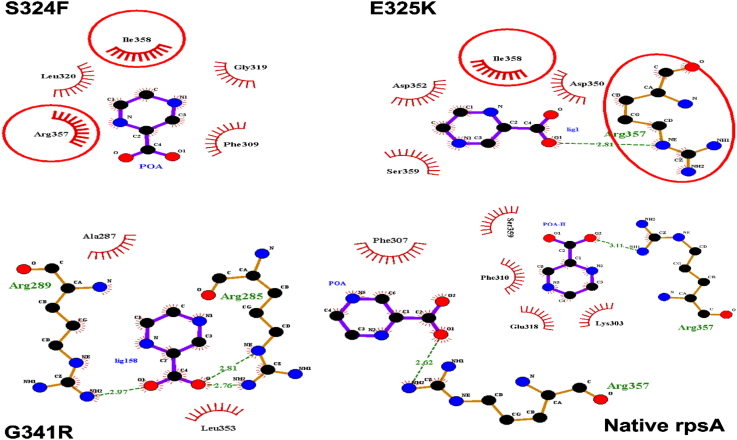
**Wild type and mutants RpsA interaction with POA. MTB RpsA domain organization. The boxes denote S1 domains. MTB**^**CTD**^**residues number (285–481) w**ild type protein formed two H-bonds and four hydrophobic with two POA molecules. Mutants S324F and E325K had fewer hydrogen and hydrophobic bonds, except for mutant G341R, which has three hydrogen bonds but only two hydrophobic interactions.

The degree of compactness and folding can be measured through radius of gyration (Rg), plotted against time. The graphs showed a variation between mutants and wild type*-*RpsA (Fig. 6). Mutant seemed to be more flexible and deviated compared to wild type protein. Variations with respect to time represent changes in folding and stability while a constant Rg value shows no change in folding during MD simulation. The plot in Fig. 3 demonstrated a degree of variation in mutants S324F, E325K, and G341R in compared to wild type. Wild type RpsA has a stable Rg value between 18.0 Å and 19 Å after 25 ns but the Rg values of mutants continuously increased during the whole simulation period.

### 3.5 Essential Dynamics of Mutants and Wild Type-RpsA Analysis

**Fig. 4:**
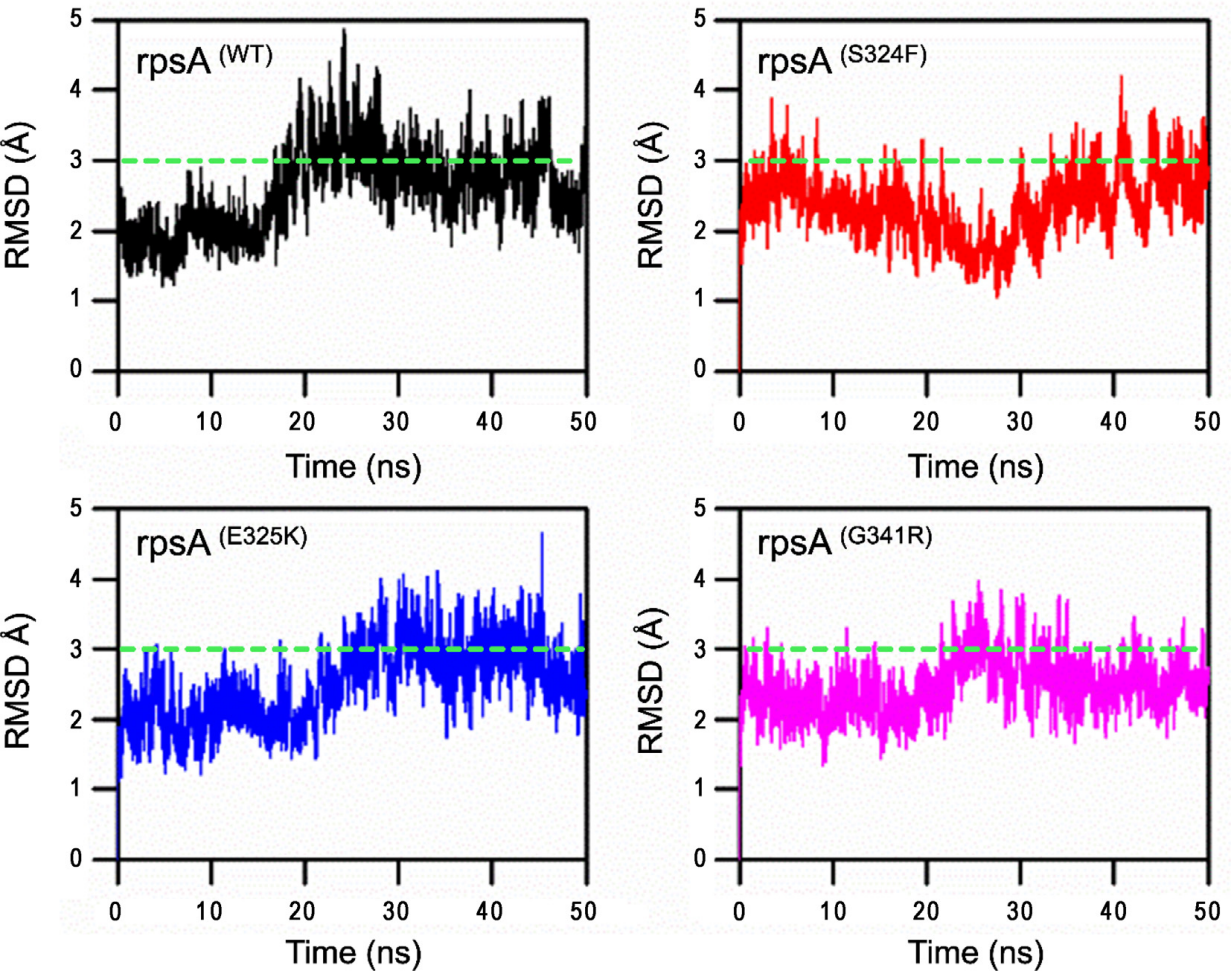
RMSD of wild type and mutants RpsA. RpsA^(WT)^; ribosomal protein S1 (RpsA) wild type had retained RMSD from 28 ns to 45 ns and dropped at 46 to 50 ns. RpsA mutant S324F RMSD seems to be inconsistent as it to be rose at 50 ns. RpsA mutant E325K also had an increased RMSD at 50 ns. RpsA mutant G341R RMSD is higher at the start at 0 ns and seems to be a little consistent but no further drop in value was seen at 50 ns.

Principle component analysis of wild typee and mutants were plotted (Fig. 7). Wild-type RpsA showed a cluster type of motion covering an area on PC1 between −188 and 140, PC2, −125 and 144, while mutants exhibited a more dispersed type of motion except E325K. S324F exhibited motion along PC1, −144 and 144, PC2, −125 and 150 (Fig. 4). However, E325K was found to be less scattered between PC1 and PC2 at −144 and 140, −75 and 75 respectively. The area of motion covered by mutant G341R along PC1 is −116 and 174, and − 75 and 125 along PC2.

The relative stability of wild type and mutants can be measured by Gibbs free energy (GFE) which is the amount of work a closed system uses when exchanging heat and work with the surroundings. The differences in Gibbs free energy values of wild type and RpsA mutants S324F, E325K, and G341R showed that mutants may alter the stability of RpsA. Wild type had a significant GFE difference with that of mutants as indicated by the peak color in the plot (Fig. 8). The peak color in both states of the wild type is more stable compared to the mutants.

The distance matrix of drug and RpsA were found to be highly deviated. The average distance was found constant throughout the simulation, however mutants exhibited a high degree of fluctuation (Fig. 9).

The total enegy measured for the wild-RpsA was significantly higher in compared to mutants. The wild type has a maximum total estimated energy of >940 kj/mol as compared with mutants throughout simulation as shown in Fig. 7. The total estimated energy of mutants G341R, S324F and E325K was measured as 880kj/mol, 937kj/mol and, 940kj/mol respectiviely.

## 4 Discussion

The emergence of first and second-line drug resistance is a major hurdle towards WHO global end TB strategy 2020. A better insight into drug resistance mechanisms is needed for better management and success of global TB erradication programs. PZA is a key first-line drug that kills subpopulations of non-replicated MTB. Previous studies reported that resistance to PZA develops due to mutations in *pncA*, but resistance may also develop due to mutations in ribosomal protein S1 (RpsA) and aspartate decarboxylase (*panD*) genes [8,[35], [36], [37]]. In our recent study, we reported some novel mutations in the RpsA gene present in PZA resistance isolates from Khyber Pakhtunkhwa, Pakistan, but the molecular mechanism behind these mutants is still to be explored. The current study provides insights into PZA resistance owing to RpsA mutations present in the conserved region associated with PZA resistance through in silico approaches. Evolutionarily conserved residues are crucial for the protein's structure and function [43]. Thus, mutations in cases of disease and resistance have been frequently found to affect conserved and surrounding sites, leading to destabilization or a loss of function [38].

Mutations in target proteins often affect flexibility and deviation, and make them a weak target for interaction with drugs. Molecular flexibility and stability fluctuations were detected from RMSF and RMSD values. Stability is an important property in increasing function and activity of biomolecules [39]. Increases in residues flexibilty of proteins may have effects on protein activity, often measured by RMSF. We found that RMSD and RMSF values of mutants S324F, E325K, and G341R were higher than wild type, affecting function and activity. These findings support the earlier studies on mutants W68R, W68G and K96R [22,40,41] (Fig. 1). Changes in protein stability, flexibility, and total energy of biomolecules have been exposed to cause loss of thermodynamic stability and protein folding [42]. The total energy of mutants may also be significantly different due to destabilization in folding and deviations (Fig. 10).

**Fig. 5:**
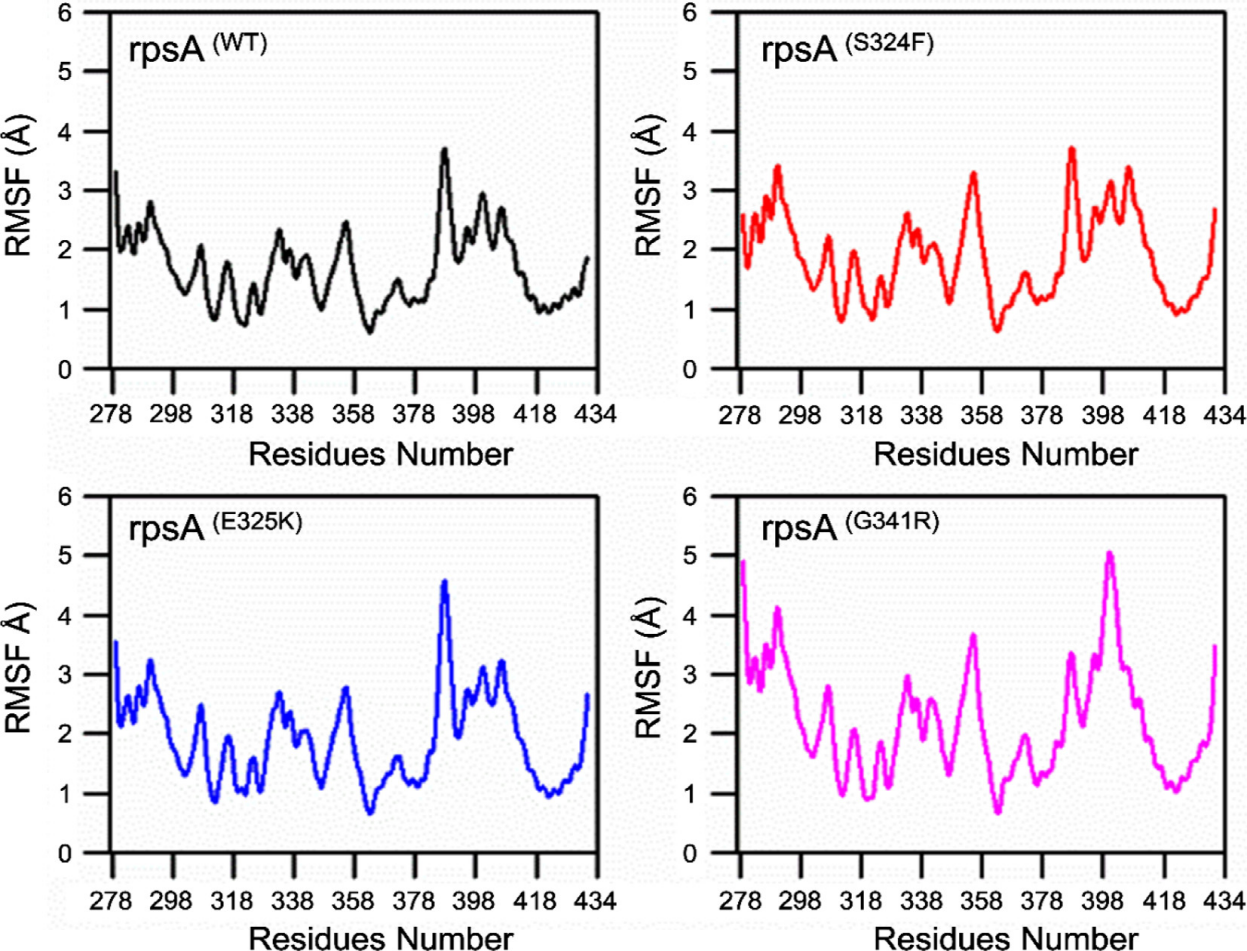
RMSF of wild type and mutant RpsA. RpsA^(WT)^; ribosomal protein S1 (RpsA) wild type had higher fluctuation in residues from 380 to 415. Mutant S324F had high fluctuation in residues 340–358 and 380–418. Mutant E325K exhibited higher fluctuation at residues 340–355 and 380–415. Mutant G341R had more flexibility at residues 320–360 and 385–400.

**Fig. 6:**
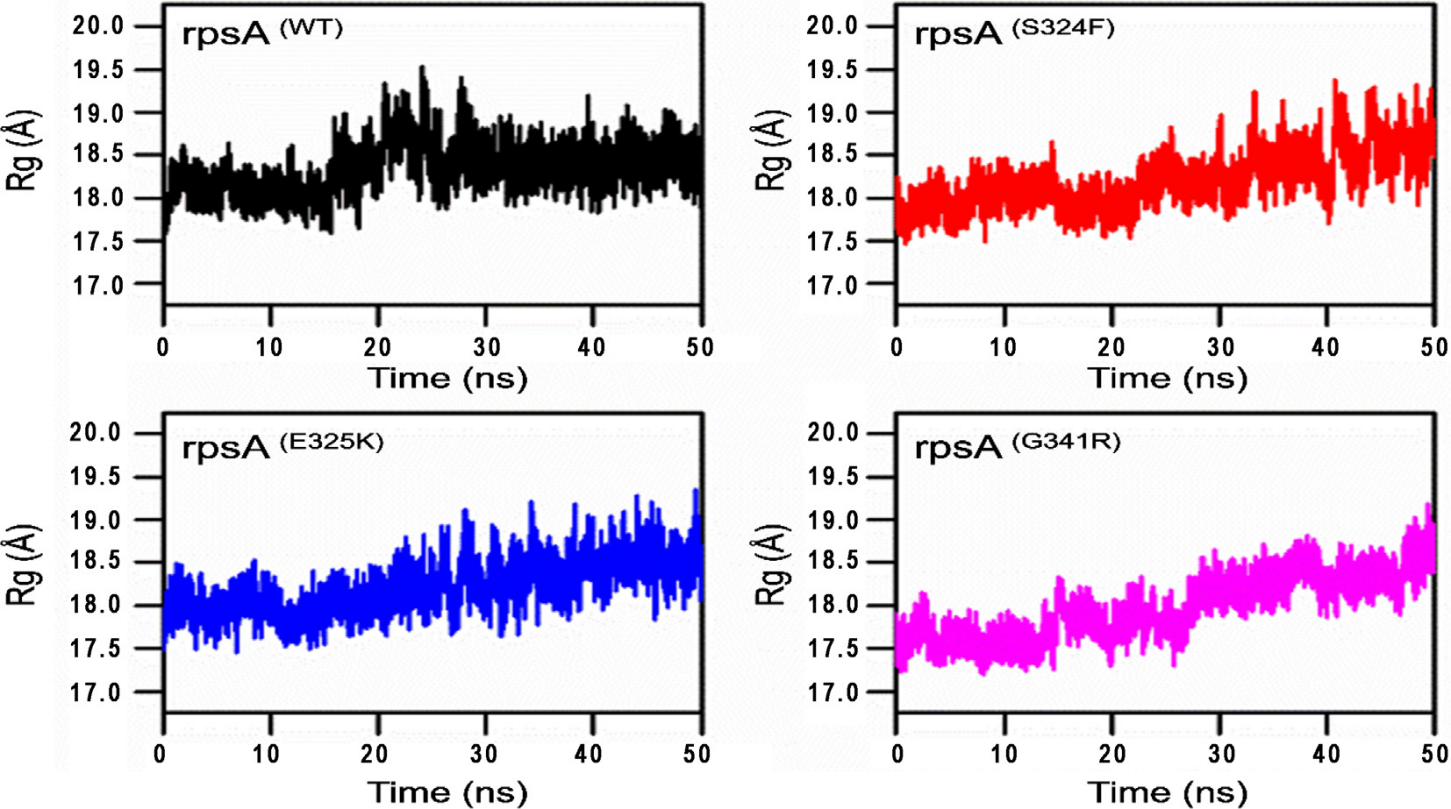
Radius of gyration of wild type and mutant RpsA. A constant Rg value shows no change in folding during MD simulation. Mutants RpsA S324F, E325K and G341R remains unstable.

Degree of compactness (a ratio of the accessible surface area of a protein to the surface area of the ideal sphere of the same volume) and folding stability was plotted against time. This degree is often measured through Radius of gyration (Rg) (Fig. 5). A stable Rg value in the simulaton period signifies the proteins folding stability, while variations in Rg suggest folding instability [43,44]. These results support the findings of Yoon et al., that mutation may have a folding effect on proteins [45]. We found a stable Rg for wild-type RpsA compared to mutants (Fig. 6), where the Rg values consistantly increased, even at 50 ns, during the entire simulation. This may be due to the abberant folding of mutant proteins. Besides folding, the pocket volume of wild type is considerd optimum as any deformation may lead to inhibited drug interaction.

**Fig. 7:**
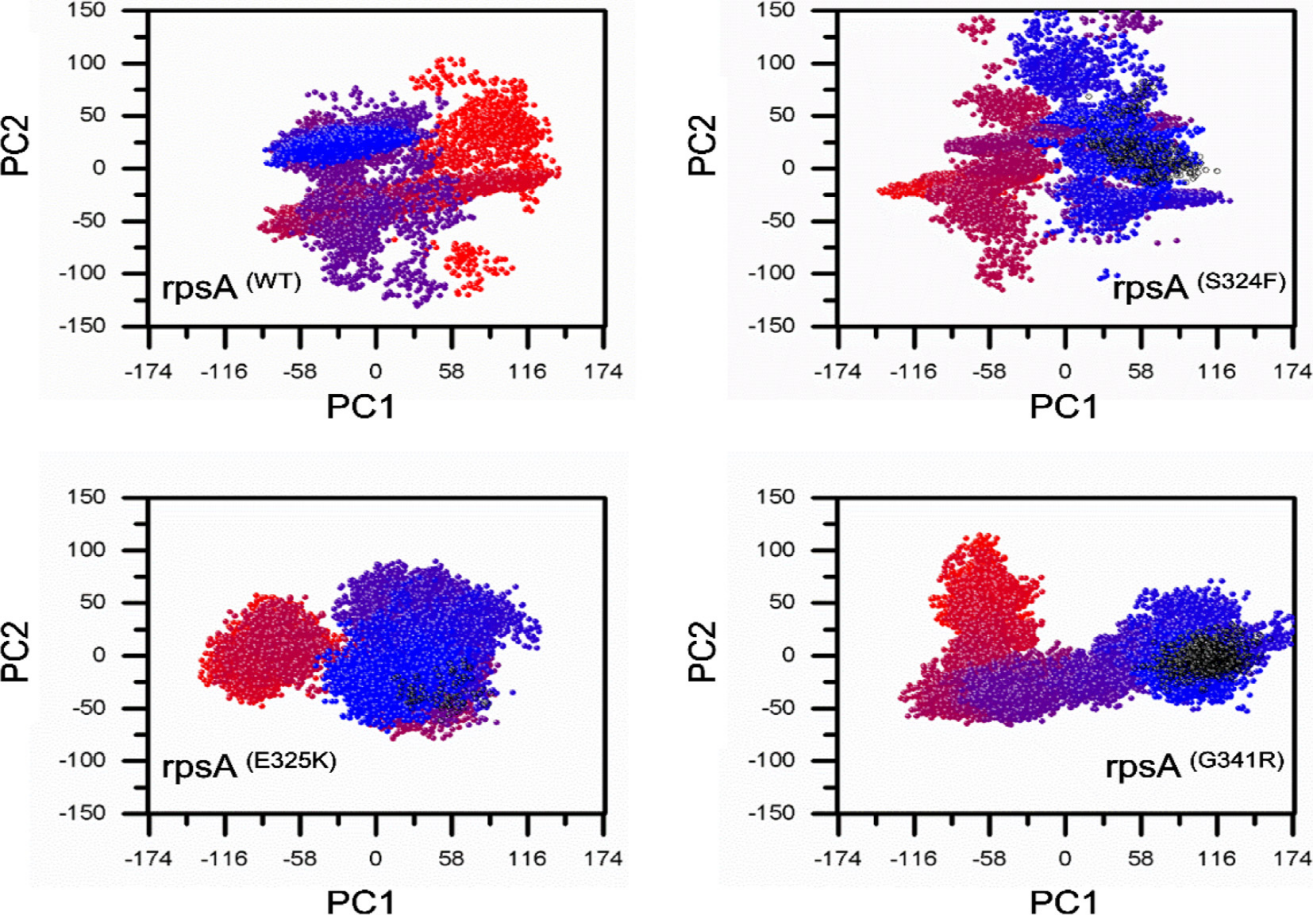
PCA of wild type and mutant RpsA. Mutants RpsA S324F, E325K and G341Rcovered a large area showing a scattered type of motion on PC1 and PC2 Gibbs free energy.

**Fig. 8:**
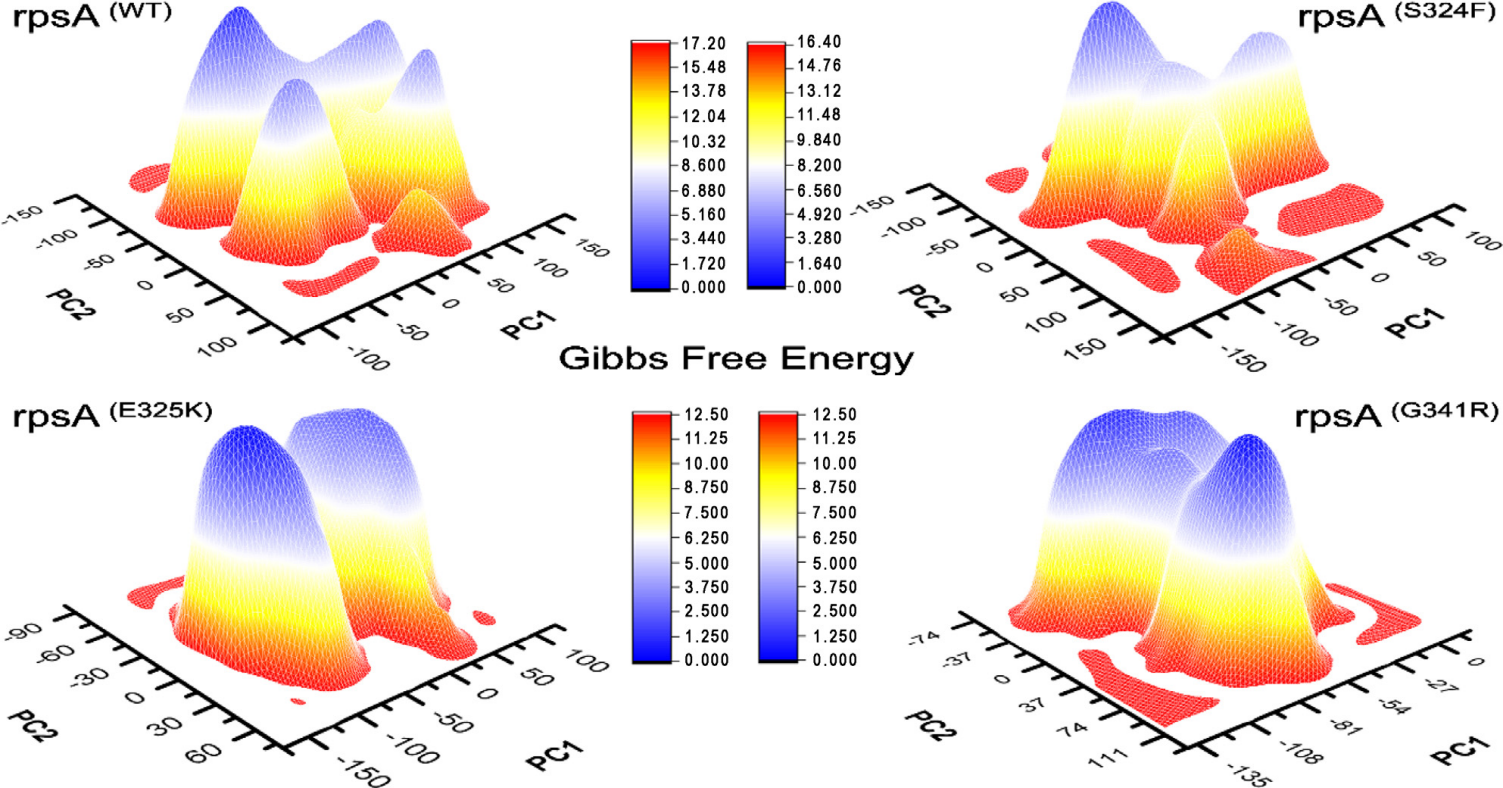
**Gibbs free energy of wild type and mutant RpsA.** Wild type has a significant GFE difference with that of mutants as indicated by the peak color of plot. The peak color of wild type is more stable in comparison to mutants.

**Fig. 9:**
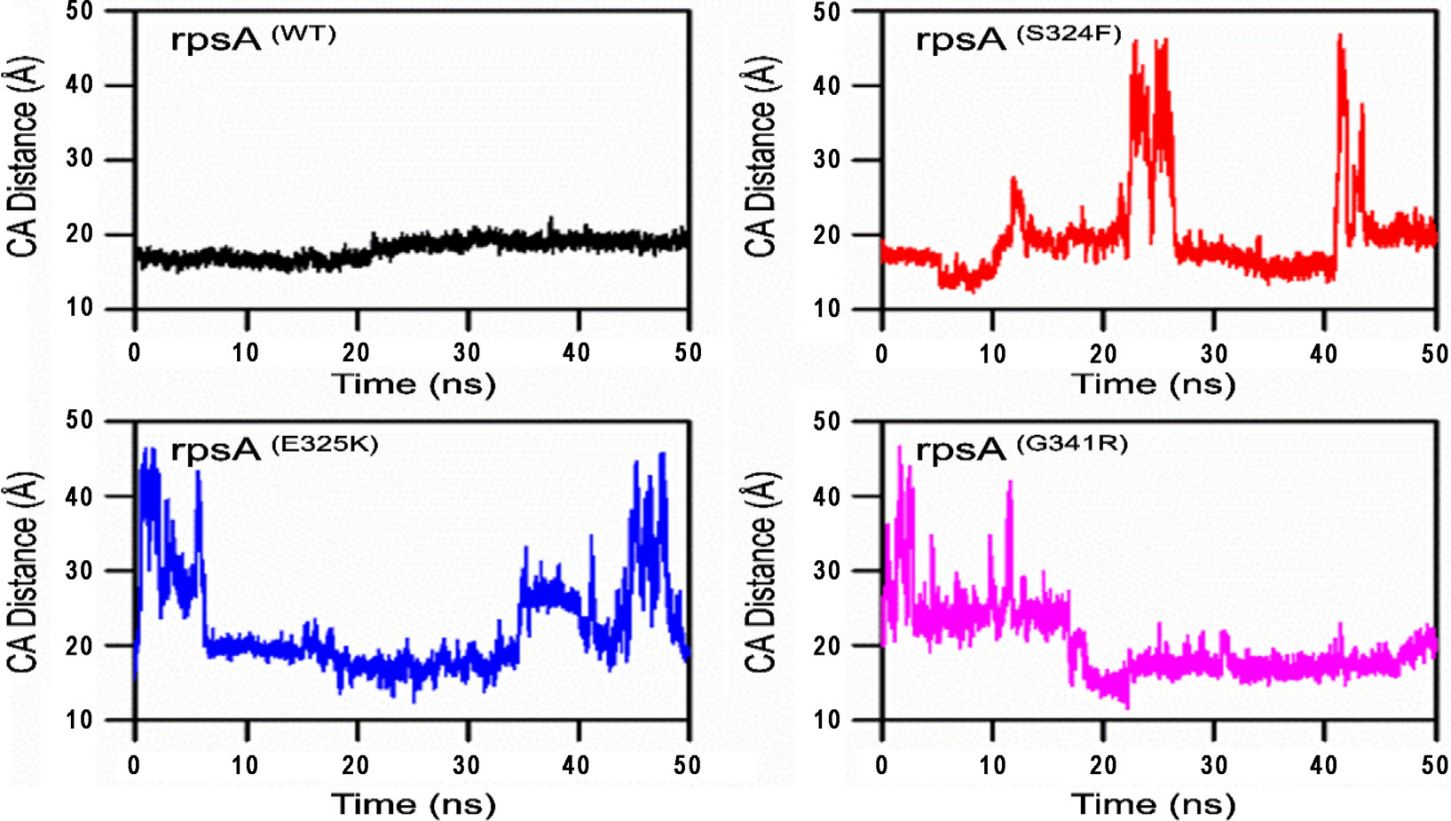
Distance matrix of wild type and mutant RpsA. The average distance of wild type and POA is constant. Mutants G341R, S324F and E325K exhibited a high degree of fluctuation.

**Fig. 10:**
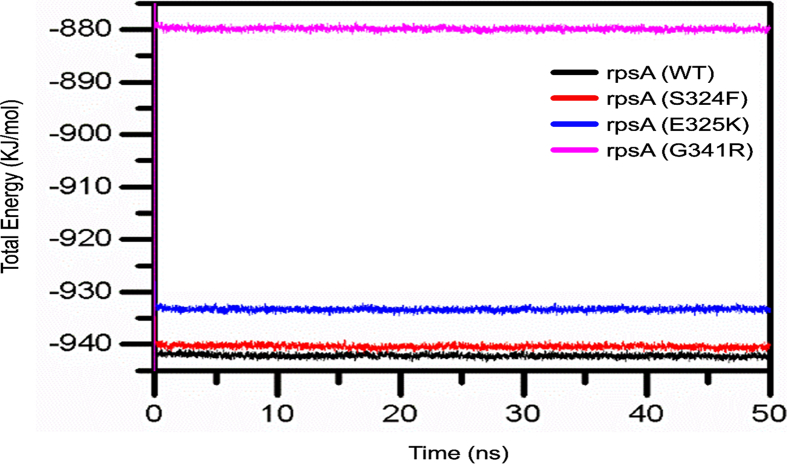
Total estimated energy of wild type and mutatnt RpsA. Total enegy measured for the wild type was significantly higher compared to mutants G341R, S324F and E325K.

A drug-binding pocket is one of the most important features of resistance and is related to size and shape of proteins [46]. Vats et al., explored the insight mechanism behind PZA resistance due to the K96R mutation, where cavity volume was significantly higher in mutants in than wild type. Binding affinity of protein to PZA may also be affected due to changes in binding pockets [41,47]. In the current study, pocket volume and shape complementarity score were found to be significantly different between wild type and mutants (Table 1). These findings suggest that resistance to POA, targeting RpsA may further be assessed from such observations.

The primary forces in protein-ligand interactions are hydrogen bonds, along with van der Waals and electrostatic forces [39]. Hydrogen and hydrophobic interactions play a key role in the three dimenstional structure of proteins, especially antibodies and enzymes. We report differences in hydrogen bonding between wild-type protein and mutants S324F, E325K, and G341R, that still offer most interactions in protein folding, stability and molecular recognition. Hydrogen bonds support the core, which is comprised of α-helix and β-sheet [[48], [49], [50]]. Both of these interactions were found greater in wild-type proteins compared with mutants. In the current study seven PZA R isolates were found as *pncA*, *rpsA,* and *panD* wild type. The possible role in resistance may be played by efflux proteins as described in a recent study [51]. The quantitative role of the efflux needs to be explored for better management of resistant TB.

In conclusion, we measured the effect of our novel mutations S324F, E325K, and G341R, and compared *rpsA* gene sequences in PZA-resistant MTB isolates with the activity of RpsA. These muations change the total energy, flexibility, stabilty and also the fluctuation of amino acid resisdues of RpsA, thereby affecting the interactions with POA. Stable interaction of drug and target is also altered due to changes in the volume of pocket of mutants S324F, E325K, and G341R, compared with wild type*-*RpsA (585.736 Å). Further, hydrogen bonding in interaction site residues and deviation and fluctuation also appeared to be affected. The overall analysis supports that mutations of RspA gene have certain roles in mediating PZA-resistance in *pncA* wild-type isolates. This is the first study of such kind where interaction between clinical mutants of RspA and POA are presented to our knowledge.

## Competing Financial Interests

All the authors have no competing financial interests.

## Authors' Contributions

SIM and DQW designed the research, MTH and SIM conducted experiments, DQW, MTH and AUR performed MD simulation and analysis, MTH, AUR and MJ wrote the manuscript.

## Funding

This study was supported by Higher Education Commission Islamabad, Pakistan under IRSIP No: **1–8/HEC/HRD/2017/8392** (http://www.hec.gov.pk/english/Pages Home.aspx) and **Dong-Qing Wei which is supported by the Key Research Area Grant 2016YFA0501703** from the Ministry of Science and Technology of China and also grants from the State Key Lab on Microbial Metabolism, and Joint Research Funds for Medical and Engineering & Scientific Research at Shanghai Jiao Tong University.
